# Axis Specification in Zebrafish Is Robust to Cell Mixing and Reveals a Regulation of Pattern Formation by Morphogenesis

**DOI:** 10.1016/j.cub.2020.05.048

**Published:** 2020-08-03

**Authors:** Timothy Fulton, Vikas Trivedi, Andrea Attardi, Kerim Anlas, Chaitanya Dingare, Alfonso Martinez Arias, Benjamin Steventon

**Affiliations:** 1 Department of Genetics, University of Cambridge, Cambridge, UK; 2 STEBICEF Department, Università degli Studi di Palermo, Palermo, Italy; 3 European Molecular Biology Laboratories (EMBL), Barcelona, Spain

**Keywords:** gastrulation, pattern emergence, explant, organiser, pescoid, hindbrain patterning

## Abstract

A fundamental question in developmental biology is how the early embryo establishes the spatial coordinate system that is later important for the organization of the embryonic body plan. Although we know a lot about the signaling and gene-regulatory networks required for this process, much less is understood about how these can operate to pattern tissues in the context of the extensive cell movements that drive gastrulation. In zebrafish, germ layer specification depends on the inheritance of maternal mRNAs [1, 2, 3], cortical rotation to generate a dorsal pole of β-catenin activity [4, 5, 6, 7, 8], and the release of Nodal signals from the yolk syncytial layer (YSL) [9, 10, 11, 12]. To determine whether germ layer specification is robust to altered cell-to-cell positioning, we separated embryonic cells from the yolk and allowed them to develop as spherical aggregates. These aggregates break symmetry autonomously to form elongated structures with an anterior-posterior pattern. Both forced reaggregation and endogenous cell mixing reveals how robust early axis specification is to spatial disruption of maternal pre-patterning. During these movements, a pole of Nodal signaling emerges that is required for explant elongation via the planar cell polarity (PCP) pathway. Blocking of PCP-dependent elongation disrupts the shaping of opposing poles of BMP and Wnt/TCF activity and the anterior-posterior patterning of neural tissue. These results lead us to suggest that embryo elongation plays a causal role in timing the exposure of cells to changes in BMP and Wnt signal activity during zebrafish gastrulation.

**Video Abstract:**

## Highlights

• Whole-zebrafish 256-cell stage embryo explants elongate
• Patterned germ layers are established
• Mesoderm formation is robust to extensive cell mixing
• Inhibition of morphogenesis blocks formation of signaling gradients

## Graphical Abstract

**Figure:**
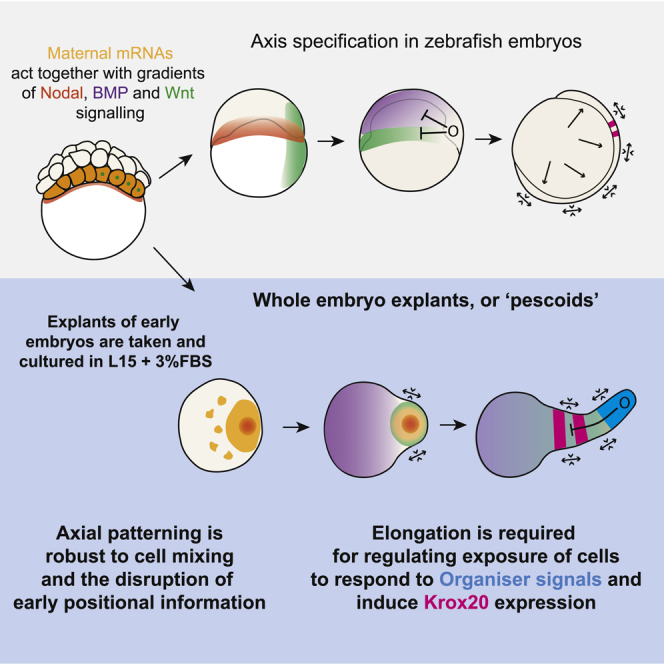

## Teaser

Fulton et al. demonstrate that, through explanting zebrafish blastomeres, termed *pescoids*, at the 256-cell stage, embryonic cells have the potential to form mesoderm, even in the absence of yolk-derived pre-pattern information caused by extensive cell mixing. Blocking explant morphogenesis results in failure to correctly pattern the neural tissue.

## Results

**Figure 1:**
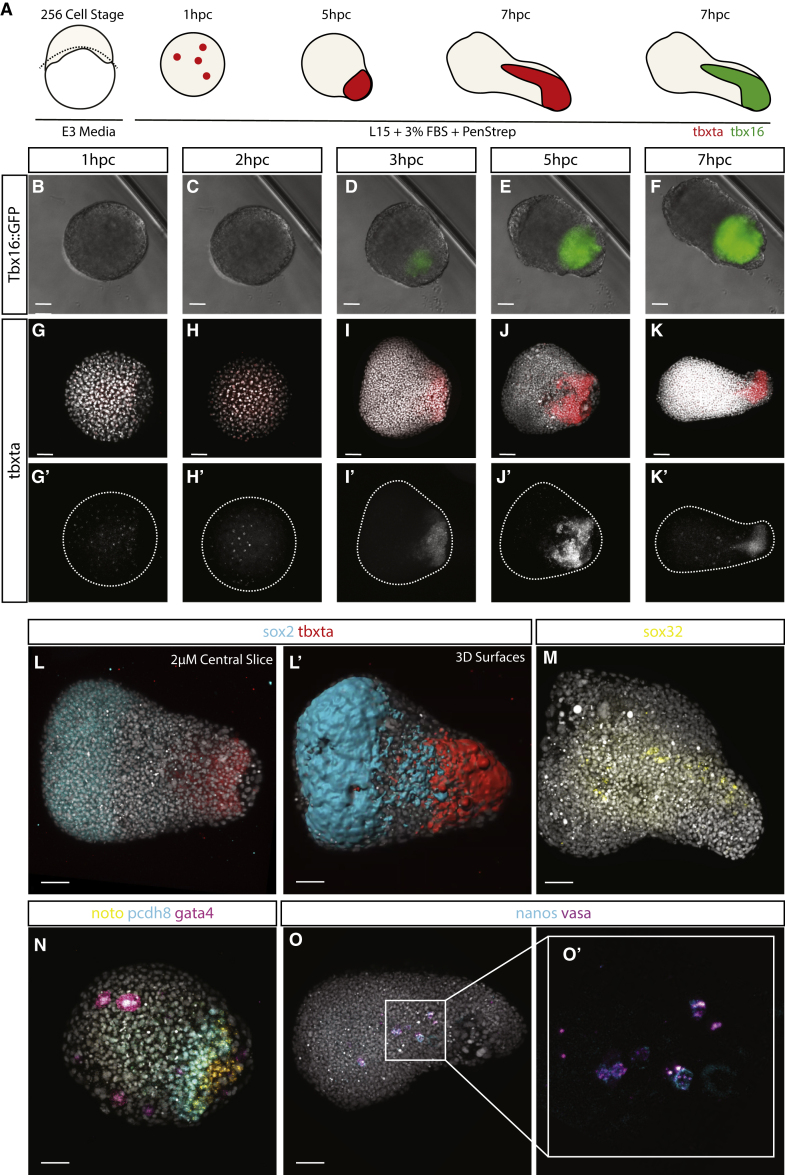
Axial Patterning Can Occur in the Absence of Yolk (A–K) Explants of early embryonic blastomeres taken at the (A) 256-cell stage demonstrate elongation and mesendodermal induction visualized through expression of a (B–F) Tbx16::GFP reporter (n = 4/8; heterozygous in-cross) and (G–K) *tbxta* mRNA (n = 1 hpc 4/4; 2 hpc 5/6; 3 hpc 5/6; 5 hpc 6/8; 7 hpc 6/6). (L and M) At the opposite pole to *tbxta* expression at 7 hpc, (L) *bmp4* expression is observed at the opposing end of the aggregate to *tbxta* expression with (M) *sox32* expression found in the center of some aggregates, but those without *sox32* expression are able to elongate (n = 3/8; *sox32* positive explants/assayed elongating explants). (N) In the high *bmp4* domain at 7 hpc, *gata4* expression is observed (n = 8/9), with protocadherin8 (n = 8/9) expression more posteriorly and *noto* furthest posterior from the *bmp4* expression domain (n = 7/9). (O) Germ cell markers (O and O’) *nanos* and *vasa* are also observed coexpressed in cells within the core of the aggregates in the *sox32*-positive domain (n = 5/5) at 7 hpc. Elongation is further quantified and shown to occur in a range of media in Figure S1. Time-lapse data of bright-field and Tbx16::GFP explants can be found in Video S1. Slice views, 3D views and surface renderings of *in situ* HCR data are available in Video S2. n = expression observed/total imaged. Scale bars represent 50 μm (A–K’) and 70 μm (L–O’).

Our current understanding of pattern formation during early development relies heavily on the notion of opposing signaling gradients that set up rudimentary body plans [13]. These gradients establish cell fates in space that in turn lead to the population-specific cell behaviors that dictate the complex cell and tissue rearrangement of gastrulation and axial elongation. In zebrafish, opposing Nodal and BMP signaling gradients are thought to be necessary and sufficient for the establishment of the body plan as shown by experiments in which deployment of such gradients in animal caps leads to the formation of a complete anterior-posterior (AP) axis [14]. In addition to controlling cell fate assignments, a recent study has demonstrated that Nodal signaling is a key driver of convergence and extension movements and is sufficient to generate these behaviors when expressed within zebrafish animal caps [15]. Furthermore, BMP levels have been shown to be important for controlling cell movements during both gastrulation [16] and posterior body elongation [13]. These observations raise the possibility that opposing BMP and Nodal signaling gradients are upstream of both morphogenesis and patterning. However, the causal relationships between these processes are difficult to untangle in situations where continuous external signaling sources are present either from the extra-embryonic signals present during early development or from overexpression experiments. To follow how cells can develop and pattern in the absence of external signals, we used primary culture of cells from zebrafish embryos prior to the formation of the zebrafish extra-embryonic yolk syncytial layer (YSL) that releases signals important for mesendodermal induction [9, 10, 11, 12] and regulation of epiboly [17]. By taking cells at the 512-cell stage and earlier, they are separated from any extra-embryonic signaling source prior to the activation of the zygotic genome at the midblastula transition (Figure 1A). These explants rapidly aggregate and round up and can therefore reveal the sufficiency of early embryonic cells to generate germ layer specification in the context of altered geometry and continued signals from the yolk and YSL.

Explants from embryos at different stages between the 64 cells and 512 cells all exhibited a similar behavior (Figure S1A): they self-organized to form polarized aggregates with a protrusion emerging from one pole. We focused our studies on 256-cell stage explants that exhibit this behavior in more than 60% of explants from each experiment (n = 32; 80–100 explants per experiment; Video S1). Quantification of aspect ratio of the longest versus shortest axis of each aggregate over time revealed a coordinated onset of elongation at 7 h post culture (hpc) (Figure S1B), demonstrating a degree of synchrony in the symmetry-breaking event. Explants from Tbx16:GFP reporter embryos [18] revealed mesoderm specification within the elongating end of the aggregate (Figures 1B–1F; Video S1), accompanied by polarized expression of *tbxta* (Figures 1G–1K). These results showed how symmetry breaking and mesoderm patterning can occur within explants of embryonic cells separated from the yolk.

Video S1. Bright-Field Time Lapse of an Elongating Explant Starting at 1 hpc Followed by an Explant Containing a Tbx16::GFP Reporter Starting at 4 hpc, Related to Figure 1 Both imaged on a widefield microscope at 10X, one frame per 10 minutes.

To demonstrate that explant elongation was not a consequence of interactions between the explants and components within the media, explants were incubated in a range of fully defined commercial media plus 3% fetal bovine serum (FBS). In L15, DMEM and PBS plus 3% FBS explants were observed to have elongated by 8 hpc (Figure S1C). Furthermore, the concentration of FBS within the culture media had minimal effect on explant elongation. Pescoids were observed elongating in L15 plus a range of FBS concentrations ranging from 1% to 10%. Again, in all these conditions, elongation was observed. Finally, to demonstrate that the presence of serum within the culture media was not the cause of elongation, pescoids were cultured in Ringer’s solution with no FBS, and elongation by 8 hpc was still observed. For the remaining experiments, we used L15 + 3% FBS as the basal media for culture.

To determine whether germ layer specification and patterning is occurring within whole-embryo explants, we stained for *sox2* as an early neural marker, *tbxta* as a mesodermal marker (Figure 1L), *sox17* (Video S2) for the early endoderm, and sox32 for the YSL (Figure 1M; Video S2). *sox2* and *tbxta* are expressed on opposing sides of the explant (Figure 1L), and surface reconstructions reveal how *tbxta*-expressing cells move into the center of the explant, although *sox2* remains superficial (Figure 1L’; Video S2). *sox32* is expressed in a band of cells at the center of the structure in 3 of 8 explants assayed with the remaining 5 explants elongating in the absence of *sox32*-positive cells (Figure 1M; Video S2). Within the mesodermal territory, a degree of additional patterning occurs with the lateral mesoderm marker *gata4* being expressed furthest from the elongating end, followed by the paraxial mesodermal marker *pcdh8* and then the notochord progenitor marker *noto* (Figure 1N). Within the *sox32* region, we find coexpression of the primordial germ cell markers *nanos* and *vasa* (Figures 1O and 1O’; Video S2). Our results demonstrate that the three primary germ layers can form and pattern in the absence of continued signals from the yolk and the YSL. In reference to similar patterns of germ layer gene expression seen in “gastruloids” from mammalian embryonic stem cells (ESCs) [19, 20], we refer to explants of early embryonic zebrafish cells as “pescoids.”

Video S2. Confocal Stacks Demonstrating the Spatial Organization of the Three Germ Layers, tbxta Mesoderm, sox2 Ectoderm, and sox17 Endoderm, within Explants, Related to Figure 1 Surface renderings of tbxta and sox2 demonstrate the spatial organistion of the ectoderm and mesoderm. This is followed by confocal Stacks and surface renderings demonstrating the spatial organization of expression of the endoderm marker *sox32*. Expression of the germ cell marker *nanos,* within the *sox32* positive domain is also demonstrated. Lastly confocal stacks demonstrating absence of HCR signal, and hence expression of Otx2. Otx2 positive control embryos also displayed.

**Figure 2:**
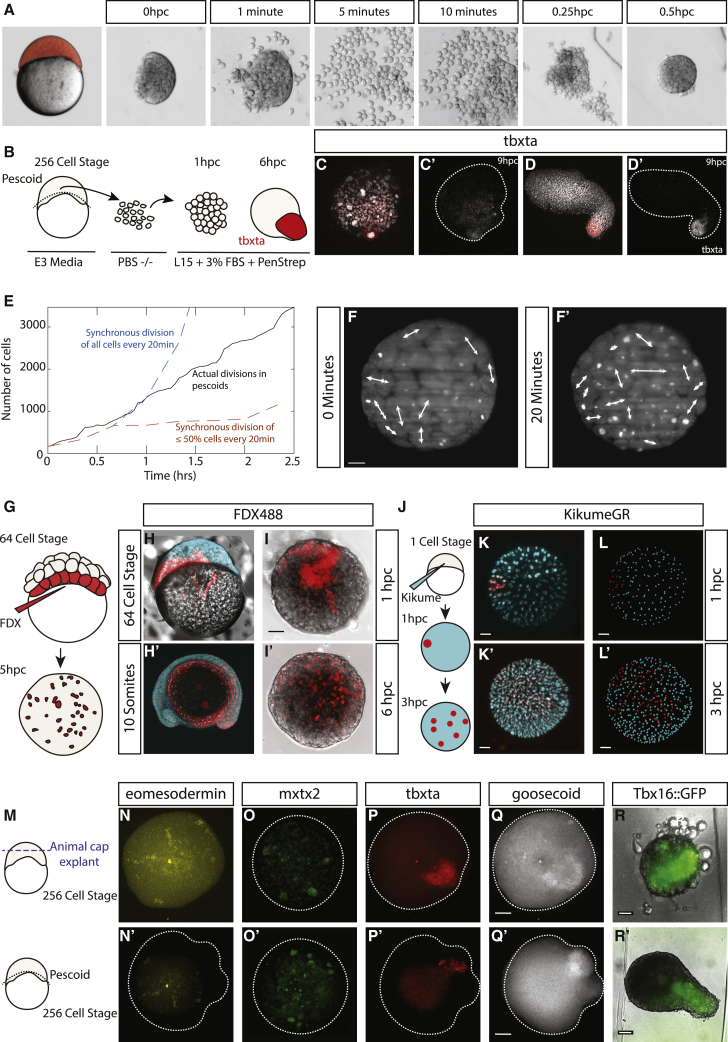
Lineage and Spatial Pre-patterns Are Lost due to Extensive Cell Divisions and Cell Mixing (A–D’) Disassociation (A) and reaggregation (B) of explanted cells results in *tbxta* expression (n = 8/8; expressed/total imaged; C and C’) and, infrequently, elongation of the aggregate (n = 2 observed; D and D’). Cells in full embryonic explants undergo rapid cell divisions as seen in images acquired on SPIM. (E–F’) Number of cells in pescoids (E) counted based on image segmentation (black curve), as seen in images acquired on SPIM (F and F’). Dashed curves are estimates of the number of cells, starting from the number of spots segmented at t = 0, if all cells divided synchronously every 20 min (blue line) or only a random sub-population (<50%) of cells divided every 20 min (orange line). (F and F’) Cells in pescoid divide randomly with no preference for direction of division, leading to mixing of cells. (G–I’) Injecting embryos with fluorescent high-molecular-weight dextran at the 64-cell stage, (H–I) labeling marginal cells prior to making pescoids demonstrates that these labeled cells spread across the entire pescoid within (H’–I’) 5 h of explanting. These cells also show a high level of intermixing of labeled and unlabeled cells. n = 10, all demonstrating cell mixing. (G–I) Images shown as maximum projections. (I’) Shown as central 2-μm slice. (J–L’) This is also shown in pescoids injected at the one-cell stage with Kikume mRNA and then a small population of cells labeled by photo conversion at 1 hpc. Explants were reimaged at 3 hpc to assay for label mixing (n = 6 replicates, all demonstrating cell mixing). (K–K’) Images are shown as maximum projections at 1 and 3 hpc. (L–L’) spot detection of labelled and unlabelled cells in 3D rendering of (K–K’) demonstating cell mixing at 1 and 3 hpc. (N–Q’) HCR staining of animal cap explants and pescoids at 5 hpc reveals a similar expression of *eomesodermin* (pescoids 4/4; animal caps 6/6), *mxtx2* (pescoids 3/5; animal caps 4/5), *tbxta* (pescoids 5/6; animal caps 3/4), and *goosecoid* (pescoids 3/6; animal caps 2/4). In (N)–(Q), n = gene expression observed/total imaged. (R) This finding is further supported by (R and R’) clear expression of a Tbx16::GFP reporter in both animal caps (4/6; expression/imaged from heterozygous in-cross) and full pescoid explant at 7 hpc (2/4; expression/imaged from heterozygous in cross). Further replicates of labeling experiments (G–L) demonstrating cell mixing are shown in Figure S2. A comparison of animal cap size explants is available in Figure S3. The SPIM data can be viewed in Video S3. Scale bar represents 50 μm.

In the embryo, mesoderm is specified in part by the inheritance of maternal mRNAs in the vegetal-most blastomeres that remain in direct contact with the yolk through the 128-cell stage [1, 2, 3] and might be important for setting up an initial symmetry-breaking event that leads to the emergence of the opposing signaling gradients described above. To test whether polarized *tbxta* expression is robust to the disruption of this early positional information, we dissociated explanted cells and reaggregated them to determine whether polarization is observed upon removal of any pre-existing asymmetry in the reaggregates (Figures 2A and 2B). In many cases, reaggregation was not complete, leading to the formation of smaller pescoids. However, in 7/10 of these reduced-sized pescoids, a polarized tip of *tbxta* expression was still observed (Figures 2C and 2C’). When most cells were reaggregated, a clear elongated morphology was observed together with *tbxta* polarization (Figures 2D and 2D’) as in non-dissociated pescoids (Figure 1L).

The ability of pescoids to undergo symmetry breaking and elongation in the absence of a pre-pattern led us to question how cells behave in early pescoids. We used light sheet imaging (selective plane illumination microscopy [SPIM]) of pescoids immediately after harvesting. Cells undergo several rounds of rapid division and increases by about 20-fold over a period of 2.5 h (Figures 2E and 2F; Video S3). In order to understand the contribution of cell division patterns to cell rearrangement in the explants, we estimated the increase in the number of cells in two scenarios: (1) if all cells were to divide synchronously every 20 min (Figure 2E, blue graph) or (2) only a random sub-population (<50%) of cells were to divide every 20 min (Figure 2E, orange graph). Compared to these estimates, it is clear that there is a degree of asynchrony in the rate of divisions across the early pescoids. Analysis of the direction of division further showed that there is no spatial pattern of divisions (Figure 2F) and, as a result of which, prior lineage-based pre-patterns would become homogenized owing to these rapid, asynchronous divisions within the pescoids before elongation. To determine whether cell rearrangements in the pescoids might contribute to the erasure of any pre-existing spatial pre-pattern, we performed labeling of marginal cells using fluorescent high-molecular-weight dextran, injected at the 64-cell stage, prior to explanting (Figures 2G–2I’). After 5 h post-culture, these labeled cells were observed distributed throughout the pescoid, with high levels of intermixing between labeled and unlabeled cells (Figures 2I’ and S2B). To demonstrate this further, small photo-labels of cells at one edge of the pescoid were made at 1 hpc and their distribution observed 3 h later (Figures 2J–2L’). In all cases, a complete mixing of labeled and unlabeled cells was observed (Figures 2K’, 2L, and S2A). These results reveal that pescoids undergo extensive cell mixing at early stages that effectively remove any pre-pattern that could be produced from the inheritance of maternal mRNAs at the vegetal pole. As a consequence, we expected that cells containing maternal mRNAs that initially reside in a vegetal domain would move to more animal regions as a consequence of this cell mixing. Indeed, hybridization chain reaction (HCR) staining of animal caps and 5-hpc pescoids revealed the presence of the maternally deposited mRNAs *eomesodermin* (Figures 2N and 2N’) and *mxtx2* (Figures 2O and 2O’), as well as expression of the early mesodermal marker *tbxta* (Figures 2P and 2P’) and marker of the embryonic shield, *goosecoid* (Figures 2Q and 2Q’) in both whole-embryo explants (pescoids) and animal caps. In addition, time-lapse movies of animal caps showed a clear expression of the Tbx16:GFP reporter (Figures 2R and 2R’). Together, these results suggested to us that the maternally inherited mesodermal specification markers are present in both vegetal and animal blastomeres by the 256-cell stage.

Video S3. Ubiquitous H2B-GFP-Labeled Explant Imaged on a SPIM with a Time Resolution of 2.5 min, Related to Figure 2

The inheritance of the maternal mRNAs *mxtx2* and *eomesodermin* to the animal pole was surprising and suggests that both animal and vegetal regions of the early blastoderm might have similar potentials to undergo elongation. When taking both animal and vegetal (Figure S3A) regions at a range of sizes, we found no bias toward vegetal explants in their ability to elongate, although tissue size was a key factor (Figure S3B). Indeed, complete pescoids elongated to a much greater extent than either poles alone, suggesting that a complete set of cells was important for pescoid elongation. To test whether this size dependency might explain the absence of elongation in animal caps that are smaller than pescoid explants, we dissociated and reaggregated 2 animal caps together. In some cases, this led to the formation of a protrusion; however, this was again dependent on aggregate size. In no cases were protrusions observed in single reaggregated animal caps (Figures S3D and S3E). This size dependency was also confirmed when cutting un-dissociated pescoids into smaller portions (Figure S3F). Individual quarters were able to express *tbxta*, but not elongate (Figure S3G). Taken together, these results suggest that aggregate size and not the gene expression state of cells within the aggregate is the major determining factor for early embryonic cells to elongate.

**Figure 3:**
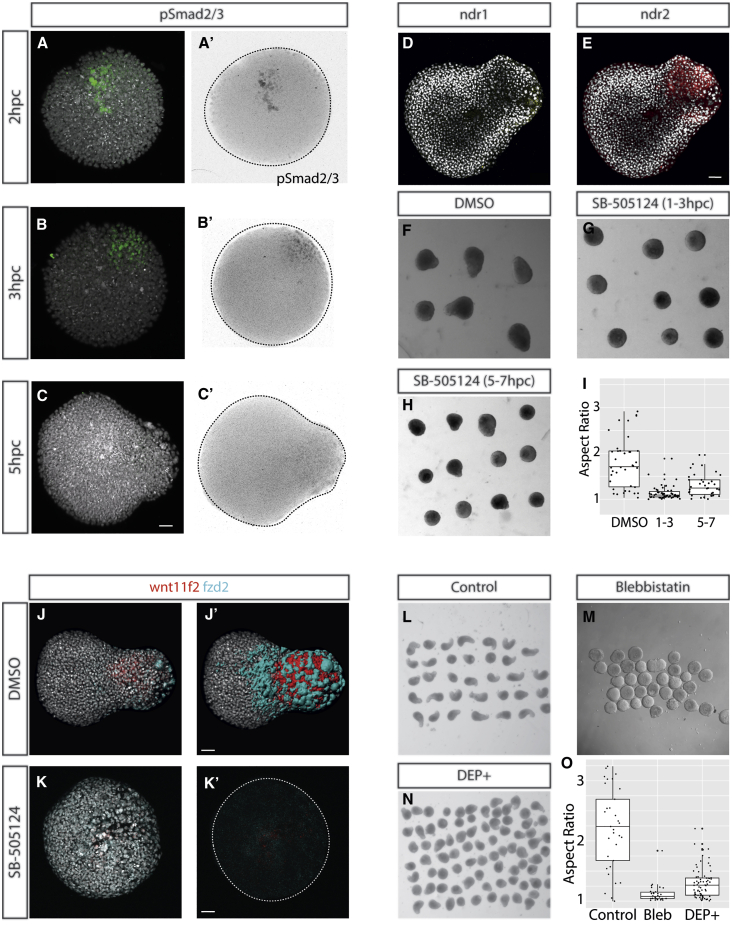
Nodal Signaling Is Upstream of PCP-Driven Convergence and Extension, which Drives Elongation (A–C) The first signaling event that polarizes to a single point within the pescoid is that of Nodal signaling, demonstrated through (A–C) polarization of phospho-Smad 2/3 activity. This is shown in composite color images and as pSmad2/3 signal inverted images (A’–C’; 2 hpc n = 4/8; 3 hpc n = 5/8; 5 hpc n = 4/6; total with polarized signal/total imaged). (D and E) We also observe polarized (D) ndr1 and (E) ndr2 expression in the elongation at 5hpc (*ndr1* n = 4/6; *ndr2* n = 4/6; expression observed/total imaged; total with polarized signal/total imaged). (F–I) Treatment with the Nodal inhibitor SB-505124 between (G) 1 and 3 hpc inhibits elongation of the explants and (H) to a lesser extent when applied between 5 and 7 hpc when compared to controls at 7 hpc (F and I). (J) The PCP components *wnt11f2* and *fzd2* are observed expressed in the elongating end of the pescoid with the spatial organization of these domains reflected in (J’) surface renderings of the HCR signal. (K and K’) Inhibition of Nodal signaling between 1 and 3 hpc results in loss of *wnt11f2* and *frz2* expression. (L–O) Inhibition of convergence and extension movements using (M) blebbistatin or (N) injection at the one-cell stage of a dominant-negative dishevelled construct blocks elongation when compared to controls (L and O), further supporting that elongation is caused by convergence and extension movements. Elongation is demonstrated not be to be caused by polarized cell division in Figure S2. Explant sizes and the ability to form mesoderm are shown in Figure S3. Polarization of Nodal signaling with the absence of polarized β-catenin or FGF (ppERK) is further shown in Figure S4. Further time-lapse data from signal reporters are available in Video S4. Scale bar represents 50 μm.

Having established that pescoids can specify mesendoderm even when cells are rearranging their initial spatial positions, we next sought to determine whether associated signaling pathways can also form polarized expression in this context. We fixed pescoids at intermediate stages between initial culture and protrusion formation at 5 hpc and stained for phospho-Smad 2/3 (Figures 3A–3C, S4A, and S4B), β-catenin (Figures S4C and S4D), and diphosphorylated ERK-1 and ERK-2 (Figure S4E). At 5 hpc, we observe an association of Smad2/3 signaling with the elongated pole is (Figure 3A), together with the expression of the Nodal ligands *ndr1* and *ndr*2 (Figures 3D and 3E). We also observe a clear association of the Nodal activity with elongation from the imaging of pescoids taken from embryos transgenic for an activin response element driving GFP expression [21] (Video S4). Furthermore, blocking Nodal receptor activity between 1 and 3 hpc with SB505124 inhibits pescoid elongation (Figures 3G and 3I), compared to controls (Figures 3F and 3I). The requirement for Nodal activity is increased at early stages as later treatments between 5 and 7 hpc have a lesser effect on elongation (Figure 3H). This importance of Nodal signaling in driving explant elongation is in line with recent work showing that Nodal signaling is sufficient to drive the elongation of animal caps [22] and an inheritance of Nodal activity from the germ ring in blastoderm explants similar to those presented here [23].

**Figure 4:**
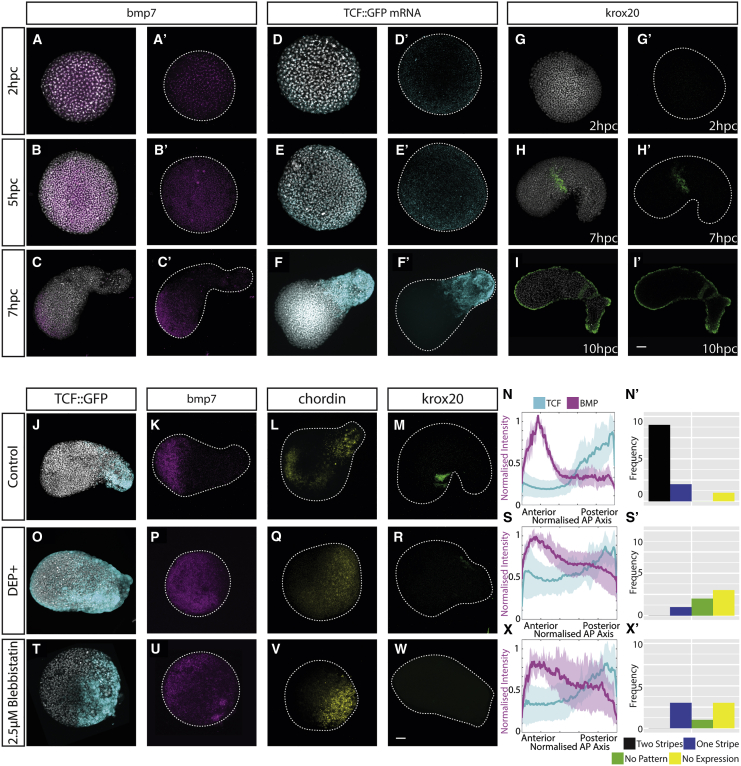
PCP-Dependent Elongation Is Required for Regulating Exposure to BMP and Wnt Activity (A–F) Expression of *bmp7* (A–C) and TCF::GFP (D–F) as a time course at 2 hpc, 5 hpc, and 7 hpc reveals both signaling domains are spread across the explant evenly at early time points and are restricted to either end of the explant by 7 hpc. TCF::GFP assayed by HCR against GFP mRNA for immediate reporter activity readout is shown (2 hpc bmp7 2/3, *tcf.:gfp* 4/6; 5 hpc *bmp7* 5/5, *tcf.:gfp* 10/10; 7 hpc *bmp7* 4/4, *tcf.:gfp* 4/5; expression observed/total imaged). (G–M, O–R, and T–W) The time course reveals the isolation of the BMP and Wnt domains to either end of the explant allows expression of hindbrain marker (G–I) *krox20* in a characteristic two-stripe pattern. (G) Initially no expression is observed (n = 0/6; expression/total imaged), followed by (H) expression of a single stripe (n = 7/8; expression/total imaged) at 7 hpc and then (I) two stripes by 10 hpc (n = 5/8 expression/total imaged). Inhibition of convergence and extension using dominant-negative dishevelled injected at the one-cell stage (O–R) and treatment with 2.5 μM blebbistatin (T–W) reveals that the Wnt/TCF and BMP domains do not separate as observed in the (J–M) control. (N, S, and X) Description of these profile quantitatively through normalization of the long axis of the explant and normalization of signal intensity between 0 and 1 (n = minimum 7 per condition; line represents mean). The lack of low BMP moderate Wnt/TCF domain can be observed in the central region of the explants compared to control. (N) displays a control profile, (S) displays the profile of a DEP+ explant, and (X) displays a profile of an explant treated with Blebbistatin. (N’, S’, and X’) The lack of low BMP moderate Wnt/TCF domains results in significantly altered patterns of *krox20* with no double-stripe patterns observed other than in controls. Scale bar represents 50 μm. Time-lapse data from signal reporters are available in Video S4.

Video S4. Bright-Field and Fluorescence Time Lapse of an Explant Containing an Activin Response Element Fusion to GFP Followed by an Explant Containing a 7xTCF-Xla.Sia:GFP Reporter, Followed by a BMP Response Element Fusion to RFP, Imaged at 10×, One Frame per 10 min, Related to Figures 3 and 4

As the PCP pathway is a well-known mediator of convergence and extension movements that drive gastrulation morphogenesis in vertebrates [24], we next sought to determine whether this is acting downstream of Nodal signaling in driving pescoid elongation. In the control situation, both the PCP ligand, Wnt11f2, and its receptor, Frizzled2, are expressed in the elongating tip (Figures 3J and 3J’). This expression is reduced upon SB-505124 treatment (Figures 3K and 3K’). Pescoid elongation is not associated with polarized proliferation, suggesting that polarized growth is not a major driver of elongation in pescoids (Figure S2C). Inhibition of the non-muscle myosin, Myosin II, with the inhibitor blebbistatin is sufficient to block elongation, suggesting that dynamic actomyosin contractions are important for this process. To assess whether this is controlled downstream of the PCP pathway, we specifically inhibited the PCP pathway using a dominant negative version of dishevelled (Dsh-DEP+) that in *Xenopus* and zebrafish inhibits axial elongation without perturbing Wnt/β-catenin activity [25]. This reduced pescoid elongation (Figures 3N and 3O), in a similar manner to that of both Nodal inhibition and blebbistatin treatments (Figure 3M). Taken together, this suggests to us that Nodal is an upstream mediator of pescoid elongation through the control of Wnt11f2 and Frizzled expression.

During gastrulation, a complex series of cell movements progressively shape the early embryo and act concomitantly with the progressive specification of cells along the anterior-posterior axis. The timing of exposure to Wnt and BMP signals is essential for patterning ectodermal derivatives [26, 27, 28, 29], yet how this temporal modulation of these signaling pathways is achieved is unknown. One possibility could be that gastrulation movements are themselves important for regulating signal exposure, by spatially separating cells expressing signals and their secreted inhibitors. Our ability to observe the progressive morphogenesis of pescoids along one primary axis of elongation offers a unique opportunity to investigate this hypothesis.

To observe how the expression and activity of BMP and canonical Wnt signaling alters during pescoid elongation, we stained explants from embryos transgenic for a TCF:::GFP reporter [30] for both *bmp7* expression and GFP mRNA at successive stages post-culture (Figures 4A–4F). Both *bmp7* and *tcf.:gfp* are uniformly expressed at low levels prior to explant elongation (Figures 4A, 4B, and 4D) but then generate opposing levels of activity at 7 hpc (Figures 4C and 4F). This correlation between elongation and the signal activity polarization can also be observed in time-lapse movies of both the TCF:GFP (Video S4) and of a BMP-responsive element driving RFP expression [21] (Video S4). This progressive polarization of Wnt and BMP activity poles also occurs with the progressive addition of bands of *krox20* expression, a marker of rhombomeres 3 and 5 [31] (Figures 4G–4I). Based on these results, we propose that pescoid elongation is important for the temporal control of BMP and Wnt exposure and for controlling the onset of *krox20* expression. To test this, we blocked elongation using either blebbistatin treatment or the injection of DEP+ and assayed the effect on *bmp7*, *chordin*, *tcf.:gfp*, and *krox20* expression (Figures 4J–4X). Neither treatment effects the level of expression of either TCF activity (Figures 4O, 4T, and 4U) or the expression of BMP pathway components (Figures 4P, 4Q, 4U, and 4V). Instead, the *tcf.:gfp* and *bmp7* expression domains are not as spatially separated as they are in the control situation (Figures 4J and 4K), meaning that a region of low *tcf.:gfp* and low bmp7 expression is no longer observed (Figures 4S and 4X). This results in a reduction of *krox20* expression (Figures 4R, 4W, and 4X) compared with controls (Figure 4M), with treated pescoids showing only one stripe of expression or less (Figures 4S’ and 4X’). These results demonstrate that elongation is an important regulator of pattern formation through the spatial and temporal regulation of BMP and Wnt signal activity.

We find that pescoid elongation is important for establishing appropriate distance from an anterior source of BMP and a posterior source of Wnt/β-catenin activity. This enables a significant proportion of tissue to be specified as hindbrain (marked by *krox20*) in a region that is low in BMP and moderate Wnt/β-catenin activity. Importantly, however, we never see *otx2* expression (Video S2), suggesting that additional spatial separation is required to create a region that is both low in Wnt/β-catenin and BMP to specify forebrain. During normal gastrulation movements, this occurs as the prechordal mesoderm moves anteriorly and continues to inhibit both BMP and Wnt activity [32]. Whether the absence of forebrain specification in pescoids is due to the lack of additional extra-embryonic signals or due to the fact that additional morphogenetic events are required to separate organizer-derived signals is an open question. The requirement for a precise temporal modulation of BMP and Wnt activity during the specification and patterning of the ectoderm is well known [26, 27, 28, 29, 33, 34]. Here, we provide evidence for a role of global tissue morphogenesis in regulating the exposure to ligands and secreted inhibitors of these pathways, potentially providing a mechanism by which patterning and morphogenesis is coordinated during gastrulation.

## Discussion

It is well known that germ layer specification in zebrafish is dependent on the inheritance of maternal mRNAs [1, 2, 3], an early dorsal pole of β-catenin activity [4, 5, 6, 7, 8], and the release of Nodal signals from the yolk syncytial layer (YSL) [9, 10, 11, 12]. That explanted cells from teleost embryos can break morphological symmetry and elongate has been known for some time [35, 36, 37]. These findings have been further confirmed recently by showing that whole-embryo explants in zebrafish also require the polarized inheritance of maternal factors from the dorsal marginal zone [23]. Here, we extend these findings to show that mesoderm specification and explant elongation can occur even when the early positional information established by these early signals is disrupted through forced dissociation and reaggregation or through the cell mixing that is occurring normally in the explants. In our hands, elongation was not observed when the cells were centrifuged following dissociation, as also reported in a similar experiment where dissociated animal cap cells were treated with activin [38] or in whole-embryo zebrafish explants [23]. This suggests that centrifugation might disrupt some component of cytoskeletal structure important for later convergence and extension movements, as suggested previously [38]. We observe a difference in the extent of mixing of labeled cells between 1 and 3 hpc (Figure 2J) compared to a similar labeling experiment of Schauer et al. [23]. However, marginal zone cells are clearly dispersed across the pescoids by 6 hpc (Figure 2G). Therefore, the cells that are local to the early polarized region of Nodal activity observed between 1 and 3 hpc will be distributed across the pescoid by the time elongation and germ layer patterning can be seen at 7 hpc (Figures 2L–2N). Despite these continual cell rearrangements, a polarized Wnt/Nodal activity can be maintained at the elongating pole of the pescoid. How this region of polarized signaling activity is maintained as cells continually move in and out of that domain is unknown. Understanding this process will be of importance as it results in the localized expression of multiple markers of the gastrula organizer, drives embryo elongation, and thereby mediates anterior-posterior patterning of the early nervous system. The phenomena of robust organizer gene expression in the context of cell movement has also been observed in chick embryos during organizer formation [39] and is likely to be a general attribute of the gastrula-stage organizer [40]. A complete understanding of this highly dynamic cellular and molecular process will require models that specifically incorporate morphogenesis in the investigation of axial patterning during early development.

Previous studies have shown that a transport and diffusion of signals from the YSL are important for ensuring a high-level precision in mesoderm specification [41]. How the diffusion of Ndr1 and Ndr2 and their inhibitor Lefty interact in the context of extensive cell mixing will be essential to obtain a complete picture of how axis specification occurs in both whole embryos and explants. During normal development, it is likely that early cell rearrangements and YSL signal release act as a precise balance to ensure that embryo patterning is both robust to alterations in external environment and the initial conditions of the fertilized egg. In the context of early embryonic explants, it is clear that the size of the explanted tissue is an important factor in allowing the tissue to polarize and elongate. Animal caps refer to explants taken of approximately 50% of the animal-most portion of the early embryo and have previously been shown to be unable to elongate *in vitro* [14, 22]. Here, we observe the same result with either small- or medium-sized animal explants failing to elongate (Figure S3C). In our hands, we do see some differences when animal cap explants are taken and cultured, as the expression of *gsc*, *tbxta*, and *tbx16* (Figure 2M) has not been observed when analyzed by colorimetric *in situ* [41]. It is possible that these differences may lie in the media used to culture animal caps, though in both cases, explants were cultured in the presence of serum that may contain additional growth factors (either 3% fetal bovine serum [here] or newborn calf serum were added) [22]. Despite these apparent differences in mesoderm specification, our results show an additional size dependency for explant elongation, as this requires either all embryonic cells to be explanted, for multiple small animal caps to be combined, or for large regions that lack either the vegetal or animal-most pole to be taken. Importantly, this size dependency for elongation can be rescued via the overexpression of Nodal activity, in PCP-dependent manner, demonstrating that these signals act together to drive convergence and extension movements [22].

The ability of embryonic cells to establish a pole of mesoderm progenitor marker expression upon dissociation and reaggregation reveals the existence of dynamic patterning process that might underpin the organization of the primary body axes in a range of species. For example, when mouse embryonic stem cells (mESCs) are allowed to aggregate in 3D to form spheres (gastruloids) in a medium that lacks spatially localized signaling cues, they display symmetry-breaking events in the absence of extra-embryonic material [19, 20]. Such behaviors resemble those that we have described here. Similar emergence of embryonic pattern has been observed in dissociated and reaggregated cells from other metazoan species, such as hydra [42], *Xenopus* [38, 43], and occurs naturally in Killifish [44]. Investigating how each of these examples differ in the precise mechanisms of axial patterning is likely to reveal further insight into how morphogenesis, morphogens, and gene-regulatory networks interact to generate pattern during complex morphogenesis.

## STAR★Methods

### Key Resources Table

**Table:** 

REAGENT or RESOURCE	SOURCE	IDENTIFIER
**Antibodies**		

anti phosophoSmad2/3	Cell Signaling Technologies	Cat#8828; RRID: AB_2631089
anti-ppERK	Sigma	Cat#M9692-200UL; RRID: AB_260729
anti beta-catenin	Sigma	Cat#C7207; RRID: AB_476865

**Chemicals, Peptides, and Recombinant Proteins**		

Leibovitz’s L15 Medium	ThermoFischer	Cat#11415049
PenStrep	Sigma	Cat#P4333-100mL
Foetal Bovine Serum	Biosera	Cat#FB-1090/500
High Molecular Weight Dextran 488nm	Sigma	Cat#46945-100MG-F
SB505124	Torcris	Cat#3263
Blebbistatin	Sigma	Cat#B0560-1MG

**Critical Commercial Assays**		

Hybridization Chain Reaction Probes and Hairpins Version 3	Molecular Instruments	N/A

**Experimental Models: Organisms/Strains**		

Zebrafish: WT (TL;AB;AB/TL)	N/A	N/A
Zebrafish: Tg(ARE::eGFP)	[21]	ZFIN: ZDB-TGCONSTRCT-160301-1
Zebrafish: Tg(BMPRE::mRFP)	[45]	ZFIN: ZDB-TGCONSTRCT-110705-4
Zebrafish: Tg(Tbx16::GFP)	[18]	ZFIN: ZDB-TGCONSTRCT-110722-1
Zebrafish: Tg(7xTCF-Xla.Siam:GFP)ia4	[30]	ZFIN: ZDB-TGCONSTRCT-110113-1

**Deposited Data**		

Raw and analyzed data	This paper	Biostudies: S-BSST410 (and on request)

### Resource Availability

#### Lead Contact

Further information and requests for resources and reagents should be directed to and will be fulfilled by the Lead Contact, Dr Benjamin Steventon (bjs57@cam.ac.uk)

#### Materials Availability

This study did not generate new unique reagents

#### Data and Code Availability

Original data in the paper is available at Biostudies:S-BSST410 and on request.

### Experimental Model and Subject Details

This research was regulated under the Animals (Scientific Procedures) Act 1986 Amendment Regulations 2012 following ethical review by the University of Cambridge Animal Welfare and Ethical Review Body (AWERB). Embryos were obtained and raised in E3 media (287.1 mg/L NaCl, 13.2 mg/L KCl, 47.85 mg/L CaCl2:2H2O, 80.685 mg/L MgCl2.6H2O and 0.1% methylene blue in 1L water) at 28°C. Wild-Type lines are either Tüpfel Long Fin (TL), AB or AB/TL. Transgenic lines for Nodal and Wnt reporters were kindly provided by the Caroline Hill laboratory [21, 45]. The Tg(7xTCF-Xla.Sia:GFP) reporter line [30] was provided by the Steven Wilson laboratory and the Tbx16::GFP reporter was obtained from the Lardelli Lab [18].

### Method Details

#### *In vitro* culture of zebrafish explants

Zebrafish embryos were incubated at 28°C in E3 media then dechorionated either manually or using pronase (1mg/mL). When embryos reached the 256 cell stage, the embryonic cells were explanted from the yolk using an eyelash tool. These tools are made by embedding an eyelash inside a glass capillary tube using agarose. Immediately after cutting, the cell mass was transferred to Leibovitz’s L15 Medium (ThermoFischer, 11415049) supplemented with 3μM PenStrep (Sigma, P4333-100mL) and 3% FBS (Biosera, FB-1090/500). Explants were incubated in this media at 28°C until fixing. A pescoid is defined as a total embryonic mass explant including all the blastomeres. When animal or vegetal pieces of differing sizes were taken, a small animal explant was taken from the animal pole of the embryo, with the remaining blastomeres being taken as labeled a large vegetal piece. When large regions from the animal pole were taken, the remaining blastomere explant was defined as a small vegetal piece. Comparison of sizes by diameter at 5hpc were made using the Fiji line drawing tool and compared to similar explants at 1hpc in [3] and 2 somite stage [15].

#### Microinjection of mRNA

Embryos obtained from the wild-type TL or AB strains were injected with 200pg of nuclear targeted Kikume mRNA (a kind gift from Ben Martin, Stony Brook University, NY, USA) at the one cell stage. Embryos were then raised to the 256 cell stage at which point they were explanted. At 0.5 hours post culture (hpc) the explant was embedded in a 1% methyl cellulose made in E3 on a glass bottomed Petri dish. About 10-20 nuclei were photo-converted from green to red using rapid exposure to 405nm laser light using a 10X air objective. The pescoid was then unmounted and allowed to grow. The pescoid was reimaged at 5hpc. Convergence and extension movements were inhibited through the injection of 300 ng/μL dominant negative Dishevelled (DEP+) mRNA at the one cell stage.

#### FDX Mosaic Labeling

Embryos constitutively expressing H2a::mCherry were injected with high molecular weight fluorescent dextran (46945-100MG-F, Sigma) into the yolk, just below the embryo at the 64 cell stage. This labels only the marginal cells. The embryos were grown to the 256 cell stage then explanted and cultured as normal. Embryos were imaged live at different stages by mounting in 1% methyl cellulose in E3 using a Leica SP8 inverted confocal with 20X air objective.

#### Pharmacological Inhibitors

To inhibit nodal signaling, explants were incubated in normal pescoid media (L15, PenStep, 3% FBS) supplemented with 20μM SB505124 (Torcris, 3263). Explants were incubated for 3 hours in treatment, or equivalent DMSO, before being washed thoroughly into normal pescoid media (L15 + 3% FBS). Cell movements were inhibited by supplementation of normal explant (L15 + 3%FBS) media with 2.5μM Blebbistatin for the entire culture period (Sigma, B0560-1MG).

#### *In situ* hybridization chain reaction (HCR)

Explants were fixed using 4% (w/v) paraformaldehyde in DEPC treated PSB. 2 pmol HCR probes were hybridized at 37°C overnight in 500μl 30% formamide hybridization buffer. This was followed by repeated washing at 37°C using 30% formamide probe wash buffer. Probes were detected by annealing of fluorescent hairpins in amplification buffer overnight at room temperature followed by repeated washing in 5X SSC 0.001% tween-20. Samples were finally counterstained using DAPI. DNA probes, fluorescent hairpins and buffers were purchased from Molecular Technologies.

#### Immunohistochemistry

Explants were fixed using 4% (w/v) paraformaldehyde in DEPC treated PSB. Explants were blocked using 3% goat serum in 0.25% Triton 1% DMSO in PBS before staining overnight at 4°C at a concentration of 1:500 in 3% goat serum 0.25% Triton 1% DMSO PBS. Secondary antibodies were incubated overnight, also at 1:500 in 3% goat serum 0.25% Triton 1% DMSO PBS, at 4°C with DAPI. Primary antibodies used were as follows – anti phosophoSmad2/3 (8828, Cell Signaling Technologies); anti-ppERK (M9692-200UL, Sigma); anti beta-catenin (C7207, Sigma). Antibodies validated against targets: anti phosophoSmad2/3 – [45]; anti-ppERK [46]; anti beta-catenin [47].

#### Fixed Sample Imaging and Analysis

Fixed explants were imaged using a #1.5 coverglass bottomed Petri dish in CUBIC clearing solution [48] and imaged using an inverted Zeiss LSM 700 confocal microscope using a 20X air objective. Confocal images are displayed without background subtraction as maximum intensity protections or 3D rendering produced using Imaris.

#### Live Imaging and Analysis

For light sheet imaging, Luxendo (Bruker) MuVi SPIM was used with samples mounted in 1% (w/v) agarose and imaged from the side. Images are displayed without background subtraction unless otherwise stated. Analysis of SPIM images was done using TGMM tracking [46]. Explants were live imaged using a wide field epi-fluorescence microscope (either Zeiss AxioObserver or Nikon, with temperature control) using a long working distance 10X air objective. Explants were free floating in narrow agarose wells to limit movement but not constrain elongation. Analysis and quantification of shape was done manually using the line drawing tool in Fiji. Brightfield images of explants under different conditions were acquired on a Leica stereo-microscope.

### Quantification and Statistical Analysis

Profiles of signal intensity were created using the line tool in Fiji set to a line width of 50 pixels. The profiles were normalized to a maximum of 1, representing the highest level of signal intensity. Profiles lengths were normalized between 0 and 1 with 0 representing anterior and 1 posterior. Samples were normalized as individuals for length and as within classes for signal intensity (ie control, treated). Profiles are displayed without background subtraction. Data were plotted using ggplot2 package of RStudio, or MATLAB. The definition of n varies between experiment and is defined in each figure legend.

## Supplemental Information

Document S1. Figures S1–S4

Document S2. Article plus Supplemental Information

Video Abstract
