# Axis Specification in Zebrafish Is Robust to Cell Mixing and Reveals a Regulation of Pattern Formation by Morphogenesis

**DOI:** 10.1016/j.cub.2020.07.022

**Published:** 2020-08-03

**Authors:** Timothy Fulton, Vikas Trivedi, Andrea Attardi, Kerim Anlas, Chaitanya Dingare, Alfonso Martinez Arias, Benjamin Steventon

(Current Biology *30*, 2984–2994.e1–e3; August 3, 2020)

Since publication, an error has been identified in Figure S1C. In the initial production of this figure, we had mistakenly duplicated the column under the heading “L15 + 3% FBS” to the left of “L15 + 10% FBS.” We have removed the second L15 + 3% FBS column to resolve this image duplication. We also duplicated images at 5 hpc L15 +3% FBS and L15 + 10% FBS. We have checked the metadata and origin of this image and confirmed it originates from the L15 + 10% FBS condition. As this error occurred in figure production and not during data analysis, this error has not impacted the conclusions of this experiment. This error has now been corrected online. The authors apologize for this error and any confusion it may have caused.

**Figure S1C:**
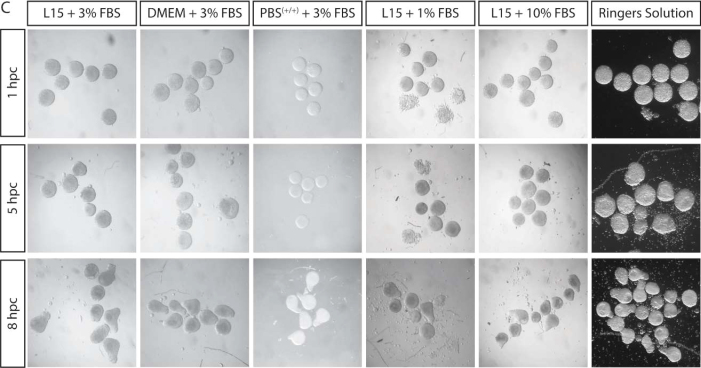
Full embryonic explants from a range of stages elongate in the absence of yolk. Related to Figure 1. (corrected)

**Figure S1C:**
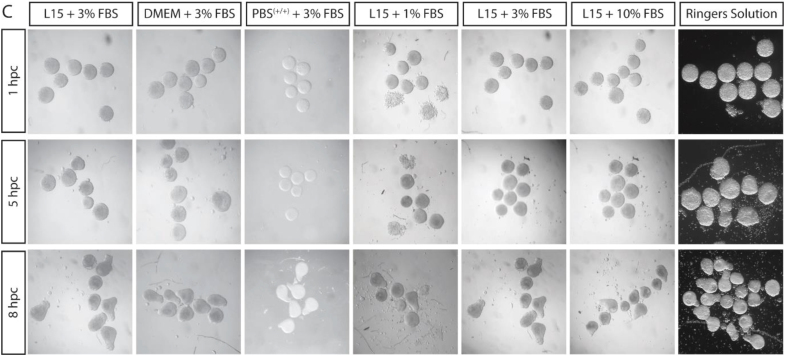
Full embryonic explants from a range of stages elongate in the absence of yolk. Related to Figure 1. (original)

