# Supplementary material for: Axis Specification in Zebrafish Is Robust to Cell Mixing and Reveals a Regulation of Pattern Formation by Morphogenesis
Source: Curr Biol. 2020 Aug 3;30(15):2984–2994.e3. doi: 10.1016/j.cub.2020.05.048 (PMC7416079; doi:10.1016/j.cub.2020.05.048)
Supplement: Document S1. Figures S1–S4 [file mmc1.pdf]

Current Biology, Volume *30*

Supplemental Information

Axis Specification in Zebrafish Is Robust to Cell Mixing and Reveals a Regulation of Pattern Formation by Morphogenesis

Timothy Fulton, Vikas Trivedi, Andrea Attardi, Kerim Anlas, Chaitanya Dingare, Alfonso Martinez Arias, and Benjamin Steventon

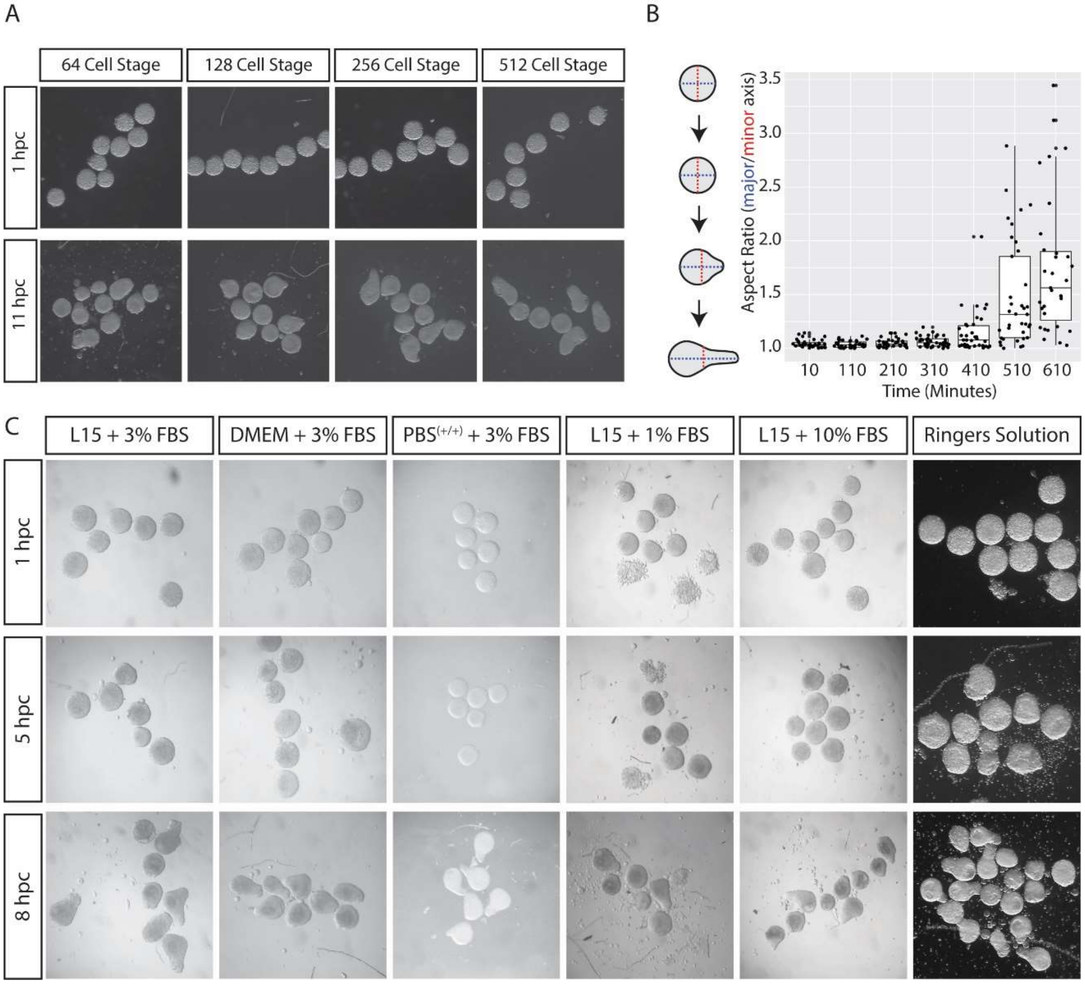

Figure S1 – Full embryonic explants from a range of stages elongate in the absence of yolk. Related to Figure 1. (**A**) Explanting the full embryo from the yolk at a range of stages between the 64 cell stage and the 512 cell stage results in spherical aggregates which elongate. (**B**) Embryonic explants from the 256 cell stage elongate, as measured by the ratio of the long axis to the short axis, over time. Elongation is pronounced after 7 hours. (**C**) Elongation in pescoids is not dependent on the culture media used, with elongation observed in a range of defined media supplemented with FBS, PBS with calcium and magnesium supplemented with FBS and Ringers Solution with no supplementation. L15 media supplemented with a range of FBS concentrations has little effect on elongation potential.

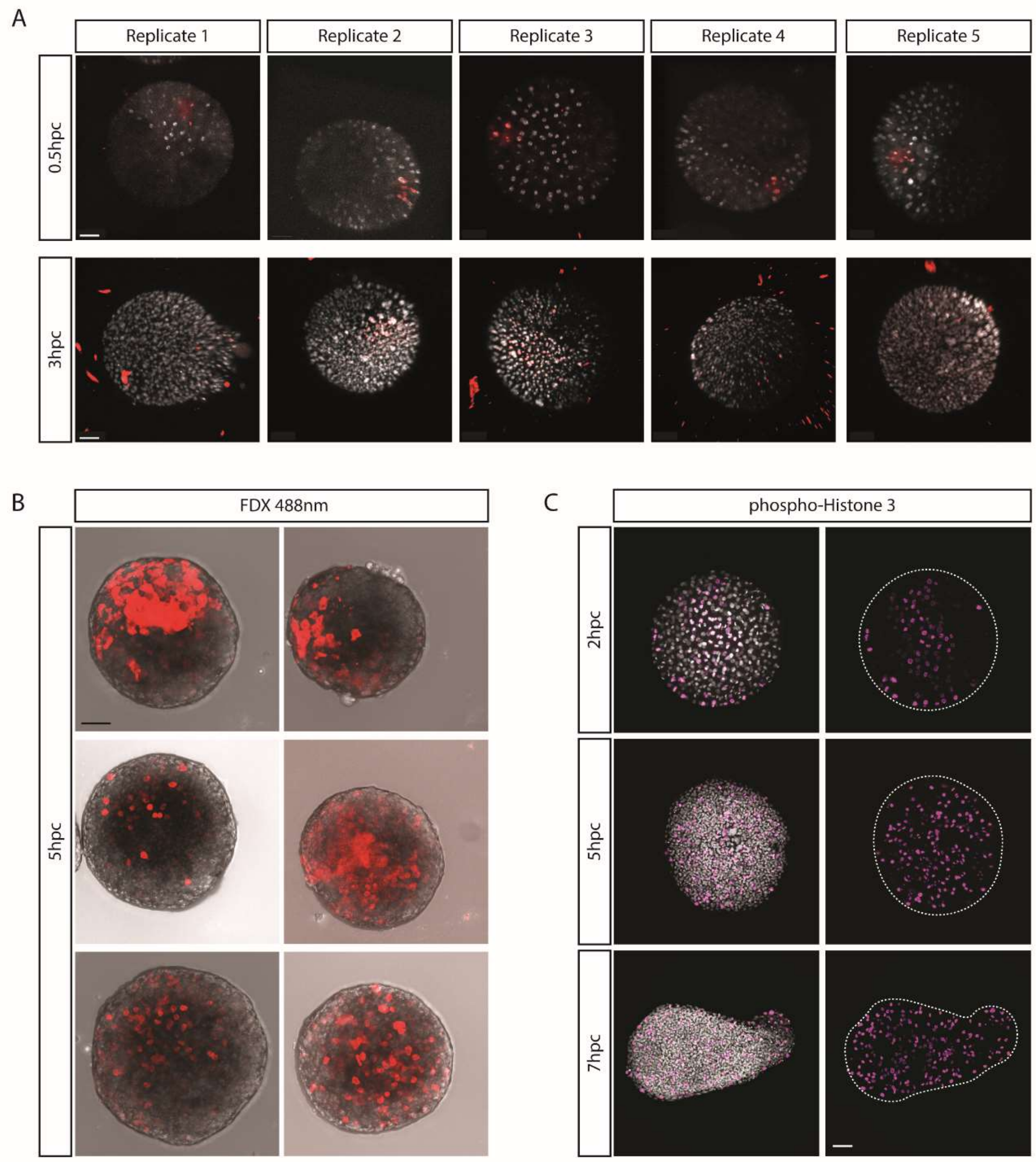

Figure S2 – Pescoids display high levels of cell mixing. Related to Figure 2 and 3. (**A**) Kikume injected pescoids are photolabelled at 0.5hpc and imaged 2.5 hours later to observe the degree of cell mixing (n= 6/6 ; explants labelled with cell mixing observed). Replicates for the experiment are shown with the labelled cells in red. Mixing is observed in all replicates. (**B**) High molecular weight dextran was injected into the 64 cell stage marginal cells to label the ventral blastomeres. Explants were taken at the 256 cell stage and cultured for 5 hours after which the degree of label spreading was assayed. Replicates are shown. (**C**) Mitotic events are not confined to one pole or region of the explants at any timepoint. Antibody stains against phosphorylated-histone H3 demonstrate high levels of cell division across the explant at all stages and with no spatial organisation (2hpc n= 6/6; 5hpc n= 9/9; 7hpc n= 6/6; Mitotic cells distributed across explant/total explants imaged). Scale = 200µm.

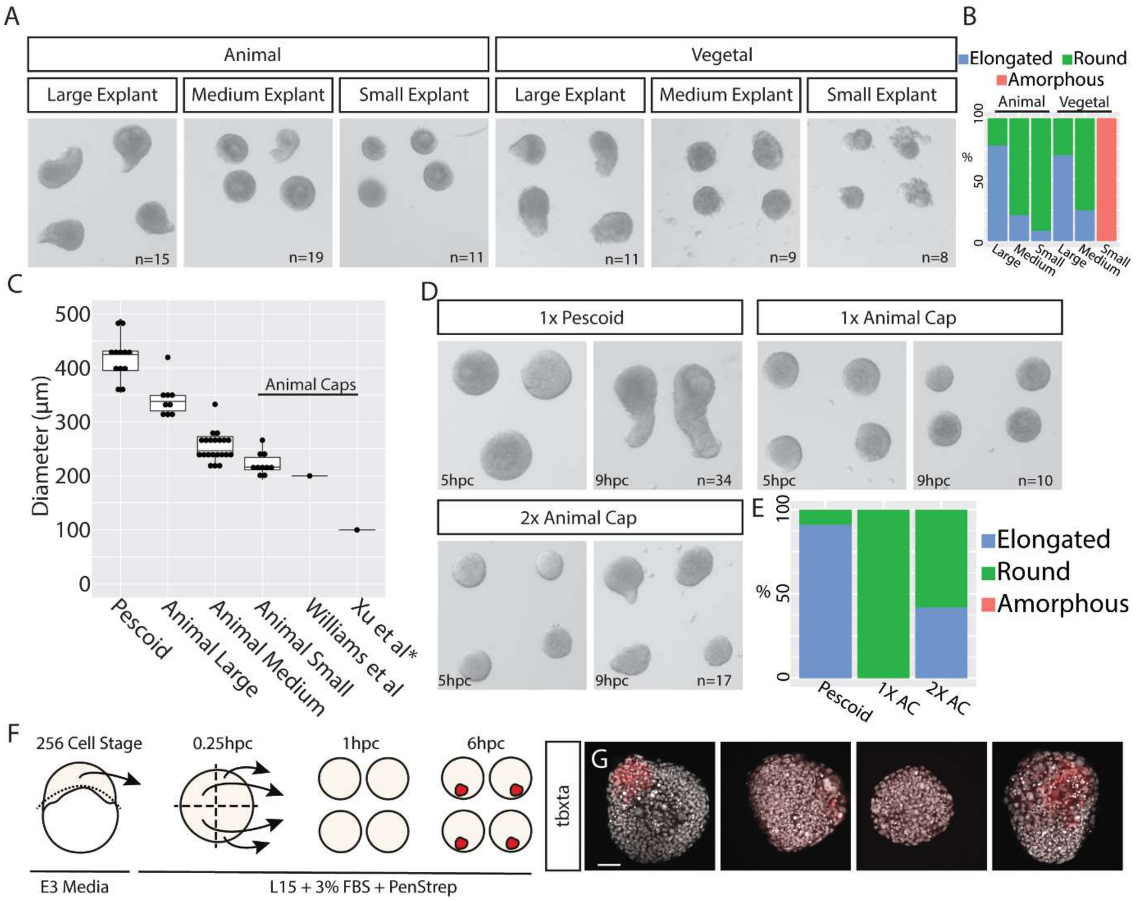

Figure S3 – Comparison of Animal Explant Sizes. Related to Figure 2. (**A-B**) Neither animal nor vegetal explants demonstrate a bias towards elongation however the size of the tissue correlates with elongation potential (n=8 explants minimum per class). After cutting small animal explants, the remainder of the embryo was explanted and classified as a large vegetal explant. After cutting large animal explants, the remainder of the embryo was explanted as a small vegetal explant. (**C**) A comparison of explant sizes to animal cap explants in Xu et al (2014) and Williams et al (2020) at 5hpc. Diameter measured from brightfield images. Xu *et al* explant measured immediately after explanting. Williams *et al* explant measured at the 2 somite stage. (**D-E**) Full pescoids demonstrate a clear and robust elongation however this is not wholly reproduced by aggregation of two animal caps together (n= 5/12 elongated/total) and was never seen in single animal cap explants (n=0/10 elongated/total). (**F**) Quartering of single pescoids also reduced the potential of pescoids to elongate however (**G**) tbxta expression was observed (n=26/32 expression observed/total imaged).

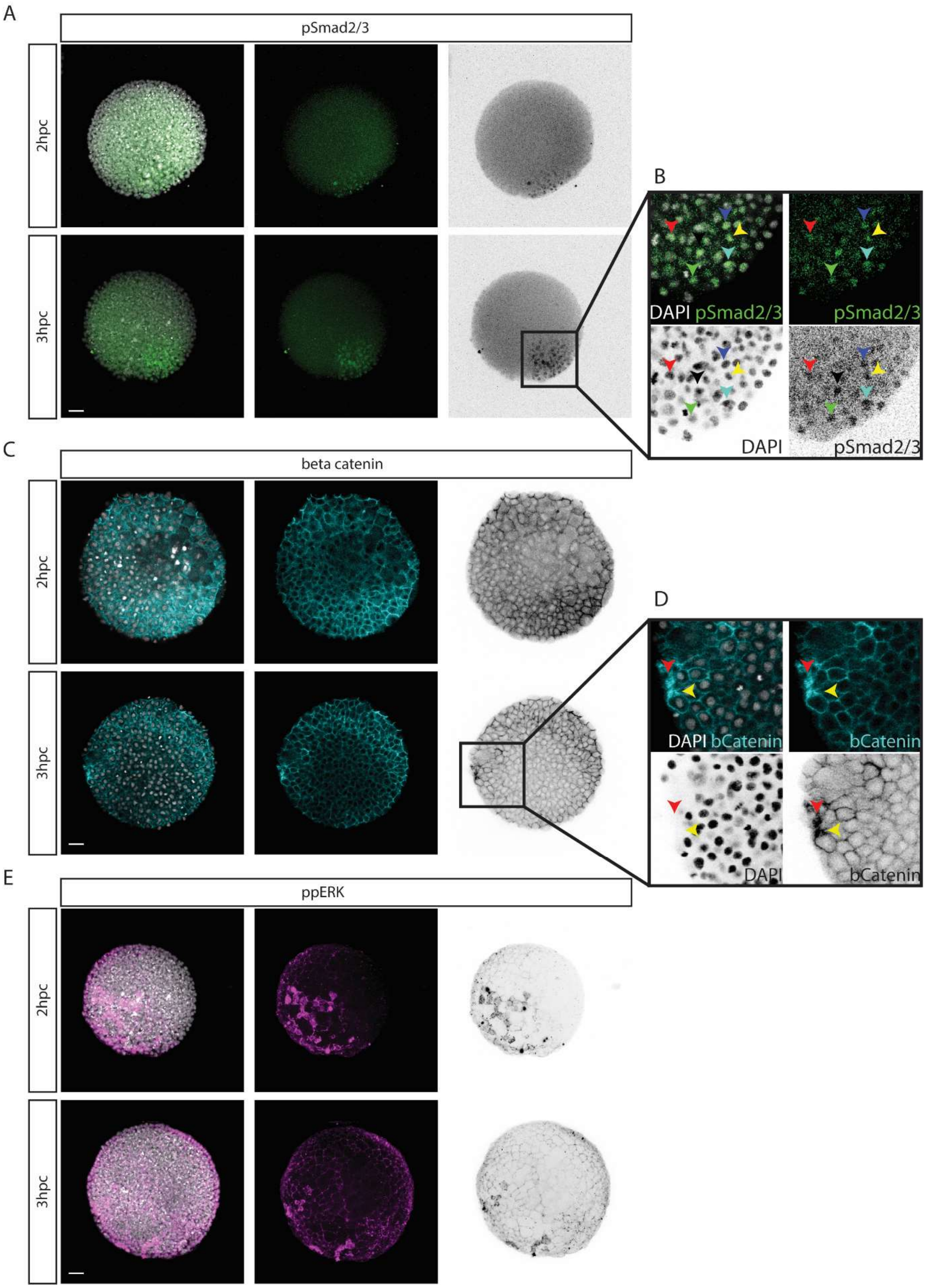

Figure S4 – Nodal Signalling is the First Observed to Polarise. Related to Figure 3. A time course of signalling markers demonstrates that (**A**) Nodal signalling measured by phosphorylated Smad2/3 is the first to polarise at 2hpc (2hpc n= 4/8, 3hpc n=5/8; explants with polarised activity/total activity). (**B**) the signal is observed

to be nuclear and displayed as merged colour images and inverted single channel images. (**C**) Wnt signalling was measured through nuclear beta catenin and after 3hpc, no nuclear signal was detected, (2hpc n= 0/4, 3hpc n=0/6; explants with polarised activity/total activity). (**D**) Regions of higher levels of signal are demonstrated to not correlate with the nuclei of cells. (**E**) FGF activity levels were measured through diphosphorylated ERK-1&2 and display no polarised activity (2hpc n= 0/8, 3hpc n=0/8; explants with polarised activity/total activity). Scale = 200μm.
